# IFN-γ, should not be ignored in SLE

**DOI:** 10.3389/fimmu.2022.954706

**Published:** 2022-08-10

**Authors:** Wenping Liu, Shumin Zhang, Jibo Wang

**Affiliations:** Department of Rheumatology & Clinical Immunology, The Affiliated Hospital of Qingdao University, Qingdao, China

**Keywords:** IFN-γ, systemic lupus erythematosus, autoimmune, biologic therapy, immune cells

## Abstract

Systemic lupus erythematosus (SLE) is a typical autoimmune disease with a complex pathogenesis and genetic predisposition. With continued understanding of this disease, it was found that SLE is related to the interferon gene signature. Most studies have emphasized the important role of IFN-α in SLE, but our previous study suggested a nonnegligible role of IFN-γ in SLE. Some scholars previously found that IFN-γ is abnormally elevated as early as before the classification of SLE and before the emergence of autoantibodies and IFN-α. Due to the large overlap between IFN-α and IFN-γ, SLE is mostly characterized by expression of the IFN-α gene after onset. Therefore, the role of IFN-γ in SLE may be underestimated. This article mainly reviews the role of IFN-γ in SLE and focuses on the nonnegligible role of IFN-γ in SLE to gain a more comprehensive understanding of the disease.

## 1 Introduction

Systemic lupus erythematosus (SLE) is a typical autoimmune disease that can affect various tissues and organs throughout the body. SLE is characterized by excessive activation of the immune system, resulting in increases in autoantibodies and immune complexes and organ dysfunction (1). At present, the pathogenesis of SLE is unclear. Sun exposure or viral infection can induce the disease in individuals with genetic susceptibilities, and women are the most vulnerable group (2). With continued development of sequencing technology, SLE was found to have a distinct interferon (IFN) gene signature (3), which is found in approximately 75% of adult patients and 90% of pediatric patients (4). Interferon is a cytokine produced in response to viral infection and has various effects, such as regulating immunity, antiviral and antitumor activities (5). According to the primary protein sequence, cognate receptor, gene locus, and cell type responsible for its production, IFNs are mainly divided into three types. Type I IFNs include IFN-α subtypes and IFN-β, -ϵ, -κ, and -ω. Type II IFNs include IFN-γ. Type III IFNs include IFN-λ (6). Many studies have shown the dominance of IFN-α in SLE (7), but some studies have also indicated that the IFN-γ gene signature may occur early in SLE (8) and may have an important role in lupus nephritis (LN) (9). Some studies found that the levels of IFN-γ and its related genes were closely related to the activation of type I IFNs in SLE patients (10, 11). More importantly, treatment against IFN-α seems to have a limited effect on SLE (12, 13), while treatment against IFN-γ could be more attractive (14). Our previous studies have emphasized the important role of IFN-γ in the initial and active stages of SLE (15). Therefore, this review will focus on IFN-γ and SLE to contribute to the understanding and treatment of SLE.

## 2 IFN-γ and its signal transduction

**Figure 1:**
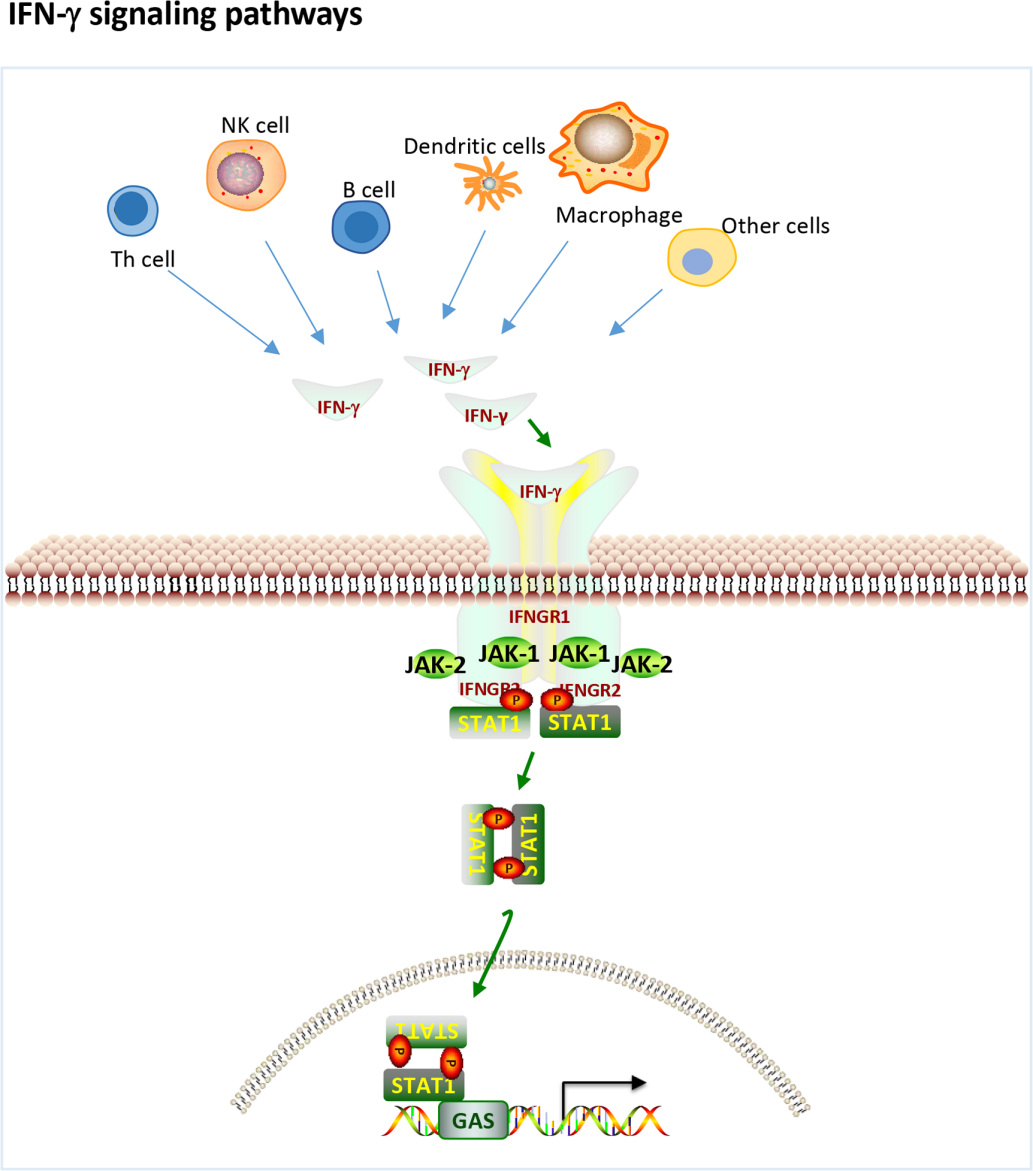
IFN-γ production and canonical signaling pathways. Th1 CD4+ T cells, cytotoxic CD8+ T cells and NK cells and to a lesser extent other cell types, such as dendritic cells (DCs), macrophages and B cells can produce IFN-γ. IFN-γ binds to the IFN-γ receptor (IFNGR) to activate JAK1 and JAK2 leading to the phosphorylation of STAT1 homodimers and binding to the IFN-gamma activation site (GAS) followed by subsequent gene transcribe.

IFN-γ is a pleiotropic type II IFN that is mainly produced by effector Th1 CD4^+^ T cells, cytotoxic CD8^+^ T cells and NK cells and to a lesser extent by other cell types, such as dendritic cells (DCs), macrophages and B cells (16). IFN-γ binds to the IFN-γ receptor (IFNGR), which is expressed on most cells and activates janus kinase 1 (JAK1) and JAK2 through the canonical pathway (**Figure 1**), leading to the phosphorylation of STAT1 homodimers and binding to the IFN-γ activation site (GAS) followed by subsequent gene transcribe (17). In addition, IFN-γ can also play a role in signal transduction through noncanonical pathways (18, 19). There is significant overlap (crosstalk) between type I and type II inducible genes, and signaling pathways can be shared between the two. Each interferon type induces the production of the other, ultimately resulting in stimulation from both sides and a mixed signature (17). Therefore, it is difficult to tell the difference between the two.

## 3 IFN-γ signature in SLE

Studies have shown that the level of IFN-γ in the serum of patients with SLE is higher than that in healthy individuals (20–23), and there is abnormal accumulation of IFN-γ in the body long before the diagnosis of SLE and before the appearance of autoantibodies and IFN-α (8). Both the mRNA and protein levels of IFN-γ were significantly higher in SLE patients than in healthy donors (9, 24), and the mRNA levels of type II IFN-inducible genes (IRF1, GBP1, CXCL9, CXCL10, and SERPING1) were elevated in SLE patients. In addition, the relative expression levels of the important transcription factors TBX21 and EOMES (25), which promote IFN-γ gene expression, were also elevated in SLE patients. We previously found an IFN-γ signature when analyzing the genetic signature of active SLE onset (15). Liu et al. evaluated the relationship between the IFN-γ signaling pathway and disease activity-related indicators and found that IFN-γ titers had good correlations with disease activity (25, 26). Manman *et al.* found that IFNG expression and the IFN-II score were positively correlated with SLEDAI scores and anti-dsDNA antibody levels but negatively correlated with serum complement third-component levels (25). Moreover, some studies also showed that the levels of IFN-γ in the serum of patients with LN were higher than those of patients with SLE without LN (27), and IFN-γ was detected in the renal tissue of patients with LN. Single-cell transcriptome analysis of kidney-infiltrating immune cells revealed that all patients produced IFN-γ (9). Furthermore, transgenic mice overexpressing IFN-γ developed autoantibodies against dsDNA and proliferative glomerulonephritis (28). Overall, the IFN-γ signaling pathway is activated in SLE patients, and IFNG levels and IFNII scores can be used as indicators of SLE disease activity to guide clinical treatment.

## 4 Genetics and epigenetics of IFN-γ in SLE

SLE is a disease with genetic characteristics. DNA sequence differences and epigenetic differences such as DNA methylation and acetylation can alter gene expression and play an important role in SLE (29). Single nucleotide polymorphisms (SNPs) are the most common genetic polymorphisms. Multiple IFN-γ related SNPs have been identified as risk loci in SLE. The greatest risk of developing SLE was detected in individuals with a Met14/Val14 genotype of IFNGR1 or a Gln64/Gln64 genotype of IFNGR2 (30). IFN-γ gene polymorphisms associated with susceptibility to SLE (31). Marut’s study found an association between the IFN-γ gene polymorphism (+874A) and the manifestations of SLE arthritis (32). A SNP of STAT4 (rs7574865) (33) was found to be associated with SLE in the IFN-STAT signaling pathway. In addition, many SNPs of interferon regulated factors (IRFs) have also been found to be associated with the risk of developing SLE, including IRF3 rs2304206 (34), IRF5 rs200464 (35), IRF7 rs1131665 (36), IRF8 rs11644034 and rs2280381 (37) polymorphism. More important, epigenetic regulation is an important mechanism of transcriptional activation in SLE pathogenesis. Epigenetics refers to the genetic regulation of changes in gene expression caused by changes in DNA methylation, histone acetylation, and chromatin accessibility without changing the nucleotide sequence of DNA (38). The enhanced response of Th1 cells in SLE is accompanied by transcriptional activation of the intracellular IFGN locus (39, 40), which is mainly due to various epigenetic changes such as H3K4 tri-methylation (41), H4-Ac catalyzed by histone acetyltransferase (42), and chromatin conformational remodeling (43). On the other hand, IFN-γ can also induce extensive remodeling of the epigenome (44). For example, IFN-γ induces IRF1-STAT1 and histone acetylation to mark promoters and enhancers of TNF and IL6 loci, resulting in increased inflammatory responses in response to subsequent over-induction by TLR ligand stimulation (45). Additionally, acetylation (H3K27Ac) is associated with gene expression, while trimethylation (H3K27me3) is associated with gene silencing (46). It was found that IFN-γ stably silences a small group of genes of anti-inflammatory including MERTK, PPARG and RANK by maintaining H3K27me3 at gene promoters (47). Thus, IFN-γ treatment made these genes refractory to the induction of glucocorticoids and IL-4. All these epigenetic changes involving IFN-γ promote and solidify the inflammation development of SLE.

## 5 Mode of action of IFN-γ in SLE

**Figure 2:**
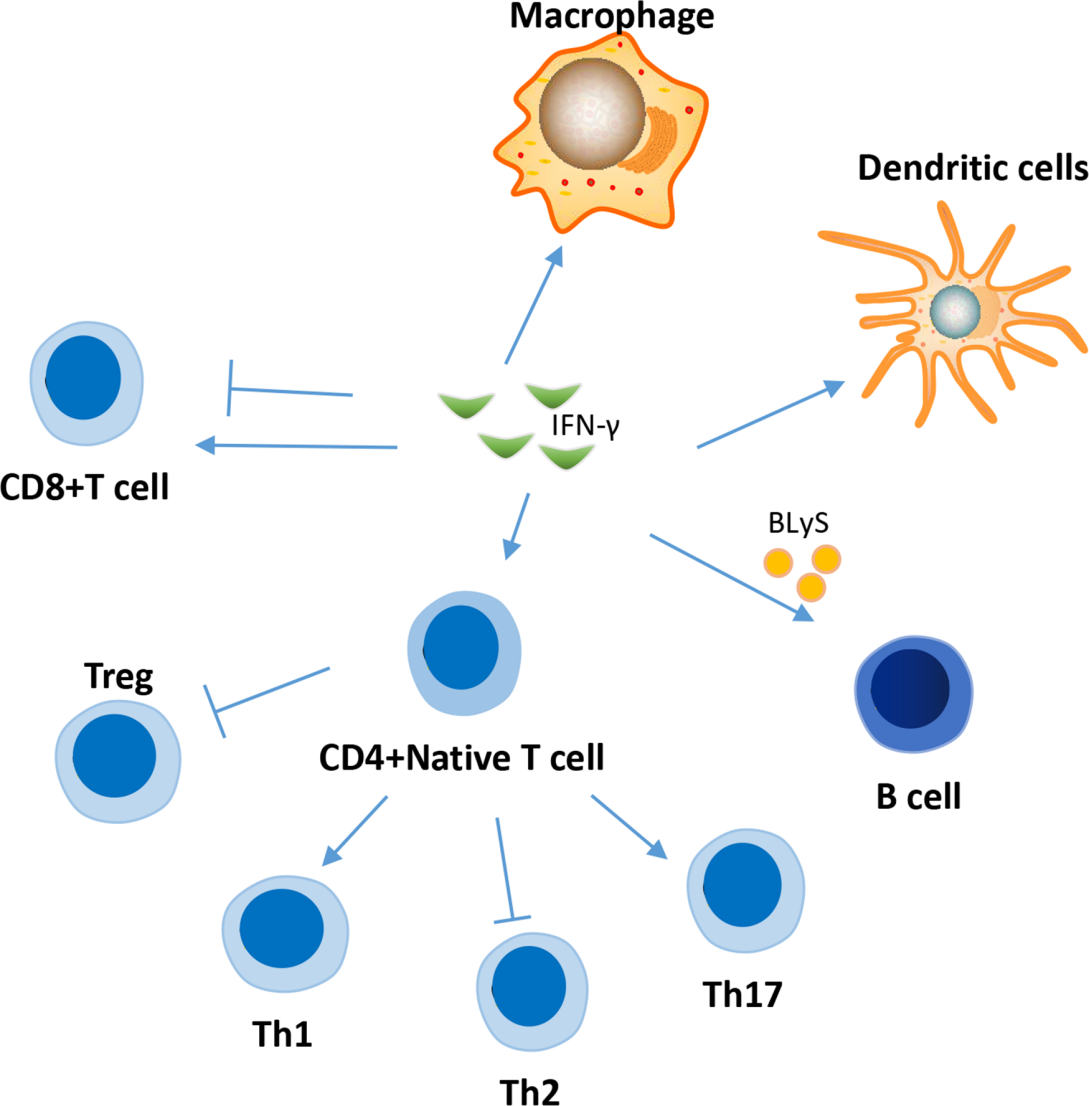
Effects of IFN-γ on several immune cells in the pathogenesis of SLE. IFN-γ affect the function of a variety of immune cells in the pathogenesis of SLE, involving T cell, B cell, macrophage, dendritic cell and et al. The effect of IFN-γ on CD8 cells in SLE is two-sided. IFN-γ can promote the differentiation of naive CD4+ T cells into inflammatory Th1 and Th17 cells, while inhibiting their differentiation into Treg cells and Th2 cells.

IFN-γ is a major proinflammatory cytokine that regulates the functions of several important immune system cells (**Figure 2**), including B cells and T cells (48, 49), and contributes significantly to the development of SLE.

### 5.1 IFN-γ affects T cell function in SLE

#### 5.1.1 CD4+T cell

Imbalance of Th1 and Th2 cells is common in the pathogenesis of SLE (50). Earlier studies suggested that the production of large amounts of antibodies in SLE was associated with Th2 responses in peripheral blood (51), but growing evidence suggests the importance of the T helper 1 (Th1) response in SLE (52). The immune response in proliferative LN has been shown to be biased toward the Th1 axis (53, 54). Th1 cells can secrete IFN-γ to promote SLE-related pathology, while IFN-γ in turn enhances the pathogenic role of Th1 cells. IFN-γ plays a vital role in the differentiation and maturation of Th1 cells (55). Although IL-12 is a typical cytokine that is necessary for the activation of Th1 cells, the role of IFN-γ is still very important (56). IFN-γ and STATI can activate the downstream transcriptional target T-bet, and the transcription factor T-bet is the main regulator of the Th1 phenotype and can stabilize the Th1 phenotype (57). More importantly, Th1 polarization in the absence of IFN-γ induction is incomplete (58, 59). In addition, IFN-γ signaling is actively involved in inhibiting CD4^+^ T-cell differentiation into Th2 (60), which is one of the reasons for the imbalance of Th1/Th2. Although *in vitro* studies have shown that IFN-γ has an inhibitory effect on Th17 cells (61, 62), increased Th17 cells and IL-17 cytokines have also been found in SLE patients (63). The ratio of Th17 and Th1 cells in SLE patients were both higher than that in healthy controls. And it was found that Th17 cells play an important role in SLE histopathological damage (64, 65). Shah’ study found that the frequency of IL-17^+^ cells was directly correlated with the frequency of IFN-γ^+^ cells (66). Anyhow, elevated IFN-γ in SLE resulting in a skewed phenotype of CD4+ T cell populations toward Th1 and Th17, which play an important role in the pathogenesis of SLE.

#### 5.1.2 Treg cell

Treg cells act as immunosuppressors, and defects in function or numbers are thought to contribute to SLE pathogenesis due to their role in maintaining peripheral immune tolerance (67). Recent studies have shown that in addition to the ability of IFN-γ to directly inhibit Treg cell function (68–70), the inhibition of effector T-cell activation by Treg cells is suppressed in an IFN-γ-enriched environment, and this inhibition requires the expression of IFNGR on Treg cells (71). Of course, there have been some studies showing that although the function and number of regulatory T cells in SLE patients are defective, this effect is due to the resistance of effector T cells to inhibit SLE, rather than defects in Treg cell functions (72). In conclusion, the mechanism by which IFN-γ inhibits Treg cell functions remains to be elucidated.

#### 5.1.3 CD8+ T cells

CD8^+^ T cells are cytotoxic cells that kill infected or damaged cells by releasing cytotoxins such as granzymes and perforin. There appears to be some inconsistency in the role of CD8^+^ T cells in SLE (73). On the one hand, CD8^+^ T cells in the peripheral blood of SLE patients often have reduced granzyme B and perforin production and exhibit impaired cytolytic function (74), which impairs the removal of autoreactive B cells and increases autoantibodies, accelerating the onset of lupus. Furthermore, the decrease in cytolytic capacity was associated with poor control of Epstein–Barr virus infection and susceptibility to infection (75, 76), which are more common in SLE. On the other hand, in contrast to the decreased cytolytic functions of circulating CD8^+^ T cells, CD8^+^ T cells extracted from sites of inflammation mostly showed enhanced effector functions (77, 78), leading to tissue damage. The effects of IFN-γ on CD8^+^ T cells are also multifaceted. IFN-γ signaling directly regulates several aspects of CD8^+^ T-cell biology. Most importantly, IFN-γ is required for cytolytic capacity of CD8^+^ T cells (79). In fact, early experiments using recombinant proteins showed that full cytolytic capacity was not achieved until CD8^+^ T cells were exposed to IFN. IFN-γ signaling in CD8^+^ T cells upregulates the expression of IL-2 receptor, the transcription factor T-bet, and granzymes. IL-2 responsiveness is critical for the generation of cytolytic CD8^+^ T cells, while granzymes are responsible for mediating the cytolysis of CD8^+^ T-cell targets (80). IFN-γ also regulates CD8^+^ T-cell proliferation after antigen exposure (81). Conversely, IFN-γ-restricted CD8^+^ T-cell effector responses were found in some studies (82). In short, the effect of IFN-γ on CD8^+^ T cells remains unclear.

### 5.2 IFN-γ affects B cell function in SLE

#### 5.2.1 B cell production

B cells play an important role in the pathogenesis of SLE (83). These cells are mediators of inflammation, enhancing inflammation and leading to direct tissue and cell damage by producing pathogenic antibodies. IFN-γ signaling can promote B-cell division during the early proliferative response following primary antigen exposure (84). IFN-γ can stimulate T cells (85), and antigen presenting cells (APCs) to produce B lymphocyte-stimulating factor (BLyS) (86), which is essential for B-cell differentiation, proliferation and survival, regulates B-cell generation and maturation (87, 88), and has been identified as a therapeutic target for SLE.

#### 5.2.2 Germinal centers formation in B cells

Furthermore, IFN-γ can induce the formation of germinal centers (GC) and B cells (89). IFN-γ integrates with BCR-, TLR- and/or CD40-dependent signaling to promote expression of the B-cell-intrinsic key transcription factor of B-cell lymphoma 6 protein (BCL-6) in mouse and human primary B cells (90). BCL-6 is critical in GC reactions (91). Lack of B-cell IFN-γR signaling significantly reduced all autoantibody isotypes by eliminating spontaneous GC formation (90). Furthermore, Chodisetti *et al.* found that type II but not type I IFN signaling was essential for TLR7-mediated promotion of autoreactive B cells and systemic autoimmunity (92). IFNγ and its downstream signaling molecules STAT1 and T-bet have nonredundant roles in B cell-mediated promotion of TLR7-driven development of AFC, GC and SLE, and type I IFN signaling contributes modestly to these processes (90, 92, 93).

#### 5.2.3 IgG class switching

In addition to IFN-γ-mediated activation of STAT1 in B cells to induce autoantibody production (89), IFN-γ plays an important role in antibody class switching. IFN-γ is able to promote B-cell IgG class switching to more pathogenic (mouse IgG2a and IgG3) autoantibodies (94–97) and promote the activation of IgGFc receptors and complement (98), contributing to disease severity. In addition, IFN-γ is involved in the development of lupus-associated hypergamma globulinemia (99, 100). IFNG also activates CD11b^+^ cells (101), enabling these cells to bind to antibody-coated target cells, thereby promoting inflammation and exacerbating the development of SLE.

### 5.3 IFN-γ affects dendritic cells

Dendritic cells (DCs) are the most typical APCs, which can activate naive T cells and trigger T cell responses that lead to tissue damage in SLE (102). Among the numerous DCs, CD11b^+^ DC subset appears to be specialized in MHC class II-mediated antigen presentation *in vivo* (103). Like the mouse CD11b^+^ DC subset, human BDCA1 DCs may act as a subset of DCs that exclusively present antigens through MHC class II molecules (104). Upregulation of CD11b^+^ DCs has been found to have a central role in the pathological development of LN and a major role in driving end-organ disease (105). IFN-γ plays a critical role in the maturation and differentiation of DCs, and affects the entire process of antigen processing and presentation. IFN-γ is considered to be an important stimulator of MHC class II gene expression (106). The ability to upregulate MHC class II is unique to IFN-γ, which induces the expression of class II transactivator (CIITA) (107), a master regulator of MHC transcription, and promotes the assembly of the MHC II enhancer. IFN-γ can upregulate the expression of CD40, CD80, CD83 and CD86 molecules on DCs to induce DCs maturation (108). Besides, IFN-γ can up-regulate the expression of immunoproteasome components of LMP1 and LMP7 (109) and the expression of transporter proteins (TAPs) associated with antigen processing (110), which plays an important role in antigen presentation process involving MHC II.

### 5.4 IFN-γ affects macrophages

Aberrant activation and unbalanced polarization of macrophages have been shown to be involved in the pathogenesis of SLE (111). IFN-γ can enhance the quantity, quality and pool of peptides bound by class I and class II MHC (106, 109, 112–114), furthermore, IFN-γ can activate the transcription of class I and class II MHC molecules, induce the expression of MHC class I and class II antigens in macrophages, endothelial cells or epithelial cells, promote the local presentation of antigens, activate macrophages (115) and induce macrophage polarization to the M1 phenotype (116, 117), which can enhance the secretion of cytokines (IL-1 and TNF) (118) and increase the release of reactive oxygen species intermediates and Nitricoxide (NO) (119). These M1 macrophages exhibit proinflammatory functions and play an important role in organ damage in SLE (120). And IFN-γ regulates the synthesis of chemokines such as CXCL10 (121). These effects have a marked effect on the activation of inflammatory cell populations.

## 6 Targeting IFN-γ in SLE

Satisfactory outcomes were observed after the application of anti-IFN-γ in a mouse lupus model (**Table 1**). Ozmen *et al.* found that treating NZB/W mice with soluble murine IFN-γ receptors inhibited the onset of glomerulonephritis (123). Werwitzke *et al.* treated lupus-prone NZB/NZW F1 mice with recombinant soluble Fc gamma receptor II (CD32), and found that it inhibited chronic murine lupus pathology*in vivo* (128). Lawson *et al.* performed intramuscular injection of a cDNA plasmid encoding IFN-γR/Fc into MRL-Fas(lpr) lupus mice, and found that lupus development and progression could be delayed, even if the treatment was initiated at a late stage (126). Besides, in (NZB)/(NZW)F1 mice, a favorable effect was observed in the treatment of lupus nephritis using an IFN-γ monoclonal antibody *in vivo* (122). Furthermore, lack of IFN-RII protects MRL/lpr mice from developing severe autoimmune-related lymphadenopathy, autoantibodies, and kidney disease (127). Deletion of the IFN-γ receptor prevents autoantibody production and glomerulonephritis in lupus-prone (NZB x NZW) F1 mice (124). Additionally, Schrott *et al.* found that chronic soluble IFN-γ receptor treatment attenuated behavioral abnormalities in autoimmune mice (125). Human clinical trials targeting IFN-γ have yielded some results (**Table 1**). AMG 811 is a fully human (IgG1) anti-IFN-γ antibody. In patients with mild to moderate SLE, a single dose of AMG 811 was well tolerated and could normalize IFN-regulated gene expression, resulting in a dose-dependent decrease in serum CXCL-10 levels (14, 129). AMG 811 treatment led to changes in IFN-γ-associated biomarkers and was well tolerated, but no significant clinical benefit was observed in patients with discoid lupus erythematosus (DLE) (130). Encouragingly positive phase Ib trials have shown the efficacy of blocking the IFN-γ pathway to treat extrarenal lupus (131). Collectively, these findings suggest that IFN-γ is a central cytokine in LN, and further studies of LN should examine IFN-γ inhibition given the acceptable safety profile of its direct blockade.

**Table 1:** Therapy effect of targeting IFN-γ on SLE.

Years	Experimental Model	Experimental Agent	Results	Ref.
1987	(NZB)/(NZW)F1 mice	IFN-γ monoclonal antibody	a favorable effect was observed in the *in vivo* treatment of lupus nephritis	(122)
1995	NZB/W mice	soluble interferon-γ receptors	inhibit the onset of glomerulonephritis	(123)
1998	(NZB x NZW) F1 mice	Deletion of the IFN-γ receptor	prevents autoantibody production and glomerulonephritis	(124)
1998	NZB x NZW F1 hybrid (B/W) mice	soluble interferon-gamma receptor	attenuated behavioral abnormalities in autoimmune mice	(125)
2000	MRL-Fas(lpr) mice	cDNA plasmid encoding IFN-γR/Fc	lupus development and progression could be delayed	(126)
2004	MRL/lpr mice	Lack of IFN-RII	protects from developing severe autoimmune-related lymphadenopathy, autoantibodies, and kidney disease	(127)
2008	NZB/NZW F1 mice	recombinant soluble Fc gamma receptor II	inhibit chronic murine lupus pathology	(128)
2015	Human SLE (Phase I studies)	AMG 811 is a fully human (IgG1) anti-IFN-γ antibody	resulting in a dose-related reduction in serum CXCL-10 levels and was well tolerated	(14)
2015	Human SLE (Phase I studies)	AMG 811 is a fully human (IgG1) anti-IFN-γ antibody	normalizes interferon-regulated gene expression and serum CXCL10 levels in patients with SLE	(129)
2017	Human DLE (Phase I studies)	AMG 811 is a fully human (IgG1) anti-IFN-γ antibody	led to changes in IFNγ-associated biomarkers and was well tolerated, but no significant clinical benefit was observed	(130)
2017	Human SLE (Phase Ib studies)	AMG 811 is a fully human (IgG1) anti-IFN-γ antibody	demonstrated favourable pharmacokinetics and acceptable safety profile but no evidence of clinical impact. IFN-γ-associated biomarkers decreased with AMG 811; effects were less pronounced and not sustained in LN subjects.	(131)

## 7 JAK inhibitors that block IFN-γ in SLE

Janus kinases (JAKs) are intracellular non-receptor tyrosine kinases that play key roles in the signaling pathways of many cytokines. This also provides a basis for the application of JAK inhibitors in the treatment of SLE. Both type I and type II interferon conduct signal transduction through the JAK-STAT signaling pathway (132), and there are many overlaps downstream of both. Therefore, blocking the JAK pathway has both therapeutic effects on both type I IFN and type II IFN mediated disease processes. Studies have found that JAKs inhibitors can inhibit the IFN signaling in human DCs, reduce CD80/CD86 expression and T cell stimulation ability (133), and reduce the production of various inflammatory cytokines including IFN-γ (134) in SLE mice. It can also restore the balance of naive CD4^+^ T cells and effector/memory cell populations in SLE mice (135). Besides, evidence from a lupus model suggests that tofacitinib (a JAK inhibitor) reduces levels of anti-dsDNA and proteinuria, and relieve symptom of nephritis and rash (136, 137). Moreover, in clinical studies, JAKs inhibitors were also found to significantly improve the signs and symptoms of active SLE, with a high remission rate of 67% for arthritis or rash in SLE patients (138, 139).

## 8 Conclusion

There are genetic features of IFN-γ in SLE, especially in the initial and active stages of the disease, suggesting that IFN-γ plays an important role in the pathogenesis of SLE. IFN-γ is an important contributor to immune regulation in the body, which may be one of the roles that cannot be ignored in the pathogenesis of SLE. Moreover, current animal studies support the feasibility of targeted IFN-γ therapy in SLE; however, no obvious effect of targeted IFN therapy has been found in human clinical trials, although some of the inflammatory indicators showed significant changes compared with those in the placebo group. Whereas, these trials are designed to focus more on drug safety than efficacy. It is worth noting that due to the important role of IFN-γ in the response to infection with some viruses, such as herpesvirus and Salmonella, during targeted IFN-γ therapy also needs to be examined in more extensive population experiments. Nevertheless, the pathogenic role of IFN-γ in SLE is of interest and treatment target IFN-γ is more promising.

## Author contributions

WL: Organize literature and original draft writing; SZ: Search literature and writing editing; JW: Conception and writing review. All authors contributed to the article and approved the submitted version.

## Acknowledgments

We gratefully thank all authors.

## Conflict of interest

The authors declare that the research was conducted in the absence of any commercial or financial relationships that could be construed as a potential conflict of interest.

## Publisher’s note

All claims expressed in this article are solely those of the authors and do not necessarily represent those of their affiliated organizations, or those of the publisher, the editors and the reviewers. Any product that may be evaluated in this article, or claim that may be made by its manufacturer, is not guaranteed or endorsed by the publisher.
